# Trends in designing microbial silage quality by biotechnological methods using lactic acid bacteria inoculants: a minireview

**DOI:** 10.1007/s11274-019-2649-2

**Published:** 2019-05-03

**Authors:** Agata U. Fabiszewska, K. J. Zielińska, B. Wróbel

**Affiliations:** 1 Department of Chemistry, Faculty of Food Sciences, Warsaw University of Life Sciences, 159 c Nowoursynowska Street, 02-787 Warsaw, Poland; 2 Department of Fermentation Technology, Prof. Wacław Dąbrowski Institute of Agricultural and Food Biotechnology, Rakowiecka 36 Street, 02-532 Warsaw, Poland; 3 Department of Grasslands, Institute of Technology and Life Sciences, Al. Hrabska 3, Falenty, 05-090 Raszyn, Poland

**Keywords:** Bacterial inoculant, Lactic acid bacteria, *Lactobacillus*, Silage, Pathogenic microflora

## Abstract

Ensiling is one of the best known method to preserve fodder. The forage before ensiling intended for silages usually contains a low number of lactic acid bacteria (LAB), so it is necessary to apply starter cultures of selected strains. Traditionally, LAB starter cultures were applied to lower the pH by producing lactic acid and to inhibit the growth of undesirable epiphytic microorganisms by competing for nutrients. Nowadays, LAB inoculants have become an effective tool for creating microbial quality of silages by selecting species with extraordinary features. Epiphytic microflora characteristic of plant material used for the production of silages and the sources of undesirable microflora in the ensiling process are discussed. This review focuses on the most frequently studied issues related to the microbial silage quality and the recent trends in increasing the quality by LAB inoculants, with respect to recent directions for selecting types of modern LAB for inoculation. Among them, the main trends described were prevention of the growth of filamentous fungi and detoxification of mycotoxins by LAB inoculants, inhibition of yeast growth by LAB present in preparations and limiting the development of pathogenic bacterial microflora through controlled fermentation with the participation of LAB and the presence of their metabolites.

## Introduction

Ensiling of raw plant material is a basic biological method for their preservation based on spontaneous lactic acid fermentation under anaerobic conditions that has been used for millennia in the preservation of roughages. Epiphytic lactic acid bacteria (LAB) utilize simple carbohydrates present in ensiled plants and metabolize it to lactic acid, and to a lesser extent to acetic acid, which prevents spoilage of the silage and allows it to be stored for a long time. The production of ensiled feed is important in countries with harsh winter seasons, where animals cannot obtain the amount of energy or nutrients they require all year round from grazing, as well as in countries with a moist climate where it is difficult to make and store hay (Pahlow et al. 2003), mostly in Europe and North America. However, it has recently gained importance in African and Asian countries as well (Mordor Intelligence Report 2017).

The composition and abundance of the epiphytic bacterial microflora on ensiled plant raw material is insufficient to initiate the production of lactic acid by the LAB present. Natural populations of LAB on plant materials are often heterofermentative and low in number (Ben-Dov et al. 2006). Obtaining silages of good quality and high digestibility requires stimulation of the ensiling process by addition of various preparations. In practice, the use of additives is recommended, especially for ensiling green fodder with low concentrations of mono-, di- and oligosaccharides and high protein content and buffer capacity. It is also recommended during cloudy and rainy weather (inability to dry plant biomass) and for ensiling plants grown in intensively amended soils. The expected changes during the ensiling process with the use of microbial additives containing LAB include the dominance of these microorganisms during the fermentation process, an increase in the ratio of lactic acid to other fermentation products (i.e., acetic acid, ethanol), faster pH decline, reduction of proteolysis and increased dry matter recovery (Pahlow et al. 2003; Muck 2013). Recently, Muck et al. (2018) published an interesting review in which authors discussed effects of silage additives, including bacterial inoculants, chemicals and enzymes in silage fermentation, aerobic stability and livestock intake and utilization. The aim of this review is to summarize the influence of beneficial microorganisms present in inoculants on microbiological silage quality and to show new trends in improving the biological quality of forages by their usage.

## Epiphytic microflora of a raw plant material intended for silages

Plant material intended for silages has a variety of aerobic and anaerobic microorganisms on its surface, referred as epiphytic microflora. Its composition depends on the type of raw material and environmental factors (weather during harvest, agrotechnics and harvesting technology). Epiphytic microflora greatly determines the fermentation of ensiled material through the amount and type of organic acids produced, at the same time affecting the stability of the obtained silage (Pahlow et al. 2003; Muck 2013).

The microflora existing on the vegetative parts of plants consists of LAB and other microorganisms undesirable from the point of view of the fermentation process and the silage quality. These include anaerobic bacilli of the genus *Clostridium*, aerobic bacteria of the genus *Bacillus*, coliform bacilli, including *Escherichia coli*,* Enterobacter* spp.,* Citrobacter* spp., *Klebsiella* spp., as well as bacteria of the genus *Listeria*,* Salmonella*,* Enterococcus* (*E. faecium*,* E. faecalis*,* E. mundtii*,* E. casseliflavus*,* E. avium*,* E. hirae*) and the occurrence of actinomycetes. Yeast and moulds also form a large group (O’Brien et al. 2005). Individual groups of microorganisms existing on plants have different requirements as to the temperature and water activity and differ in the possibility of using organic compounds. The increase in the number of epiphytic microflora is favoured by long drying of meadow grass before harvest, high dry matter content ( > 40%), occurrence of rain or high temperatures during drying, contamination of forage with soil and use of high doses of natural fertilizers (Pauly and Rodhe 2002).

Epiphytic LAB are essential for spontaneous silage fermentation and are a relatively small group of microorganisms, not exceeding one percent of the total microflora in plants. There is a big variety in numbers of LAB on the crops, from the lower detection limit of 10  CFU/g on alfalfa up to 1 × 10^7^ CFU/g on sorghum and maize (CFU: colony forming units). Cool weather lowers the numbers of LAB, and high amounts of LAB therefore occurs often at second and third harvests, when the temperature is at its highest in climates like northern Europe for grasses and alfalfa and for early cultivars of maize (Pahlow et al. 2003; Broberg et al. 2007; Comino et al. 2014).

Dominant species involved in the fermentation process are bacteria of the genera *Lactobacillus*,* Pediococcus* and *Lactococcus*. The LABs of the genus *Lactobacillus* (*L. plantarum*,* L. brevis*,* L. casei*,* L. rhamnosus*,* L. curvatus*,* L. gasseri*,* L. pentosus*) do not exceed 1 × 10^3^ CFU/g. The presence of bacteria of the genus *Pediococcus* (*P. pentosaceus*,* P. acidilactici*,* P. damnosus*,* P. confusa*) has been observed at a number that does not exceed 1 × 10^3^ CFU/g (Müller et al. 2001).

New polymerase chain reaction (PCR)-based techniques are uncovering some new species of epiphytic microflora on plants (Muck 2013). Brusetti et al. (2006) reported the presence of *B. megaterium* in the ensiling of maize, as well as *Weissella kimchii* and *Enterococcus flavescens*. New species that have been isolated from silage include *Lactobacillus taiwanensis* (Wang et al. 2009) and *P. lolii* (Doi et al. 2009). Pang et al. (2011a, b) reported the presence of *Leuconostoc lactis*, *E. mundtii* and *W. cibaria*. The same group isolated LAB strains from maize, rice, sorghum and alfalfa silages and found that *W. cibaria* and *W. confusa* were the dominant species observed in maize silage.

## Undesirable microflora and its sources in plant material intended for ensiling

The ensiling process in feed is accompanied by the fermentation of sugars that lead to a decrease in pH and the growth of undesirable microorganisms, such as yeasts and moulds (O’Brien et al. 2007). Silages, however, are a matrix in which mould growth can occur, even in the last days of the shelf-life of silages (Richard et al. 2007). The filamentous fungi are microorganisms that often cause spoilage of roughages (Magnusson and Schnurer 2005).

Many mould species are also capable of producing secondary metabolites that toxic to human and animals, called mycotoxins, the presence of which in feed evoke a threat to food safety (Van Egmond 2004). Silages in Europe, Canada and Australia can be contaminated with toxins produced by moulds of the genus *Penicillium*, e.g., *P. verrucosum*, and some *Aspergillus* species, e.g., *A. ochraceus* and *A. sulphureus*, which can produce ochratoxin A (OTA) and aflatoxins (AFs; Richard et al. 2007). Research on microflora of silages made of maize showed that the most common species of potentially toxic moulds are *Arthrinium phaeospermum*, *Aspergillus* sp., *Byssochlamys* spp., *Fusarium* spp., *Monascus ruber* and *Penicillium* spp. (O’Brien et al. 2006).

Mycotoxins like aflatoxin B_1_ (AFB_1_) present in forages are accumulated by cows and excreted into milk. Due to the carry-over effect, contaminated milk may be a source of mycotoxins for people consuming toxin-contaminated products. Under European climatic conditions, AFs can be nested, for example in certain layers of silos heated by the sun or in hot hay. The presence of mycotoxins in livestock is still a problem, especially as there is no monitoring of the level of contamination (Coffey et al. 2009; Dalie et al. 2010; Oliveira et al. 2013; Rubert et al. 2014).

On farms, especially organic ones, the threat of microbial contamination of plant material that comes from organically fertilized soils, for example not fully digested liquid manure, is real. Faecal bacteria present in organic fertilizers, e.g., the bacteria *Salmonella* spp. or *E. coli*, after introduction into the soil or applied to growing plants can survive in the soil and be present in the plant material (Ongeng et al. 2015). The survival time of *Salmonella* spp. in the soil depends on the conditions and can last about 33 weeks or longer. The reduction of faecal bacteria in the soil varies due to the temperature, pH, type and humidity of the soil, as well as the season and presence of an antagonistic microflora in relation to pathogens (You et al. 2006; Holley et al. 2006; Edrington et al. 2009).

## Inoculants of lactic acid bacteria

Bacterial inoculants for silages can be divided into one-strain preparations and multiple-strain preparations. Moreover, the inoculants can be supplemented with enzymes such as xylanases and betaglucanases, which aim to enhance the availability of monosaccharides and disaccharides for bacterial cells.

Most commercially available inoculants contain homofermentative LABs, which are fast and efficient producers of lactic acid, and thus improve the silage fermentation. Among the most popular species are *L. plantarum*, *L. acidophilus*,* E. faecium*, *P. acidilactici* and *P. pentosaceus*. Due to the ability to produce volatile fatty acids (e.g., acetic acid), heterofermentative species are sometimes included in silage starter cultures (Weinberg et al. 2003). For several years, other abilities of LAB species were considered for their presence in silage inoculants (Table 1).

**Table 1:** Role of beneficial lactic acid bacteria species present in inoculants on microbiological silage quality

Genus of LAB	Strains of species LAB	Characteristics	References
*Lactobacillus*	*L. plantarum*	Dynamic increase of population in silage, antibacterial and antifungal activity, decontamination of mycotoxins	Luz et al. (2018), Guo et al. (2018) and Zielińska et al. (2014)
	*L. casei*	Probiotic bacteria, antibacterial and antifungal activity, decontamination of mycotoxins	Vinderola and Ritieni (2015) and Luz et al. (2018)
	*L. rhamnosus*	Probiotic bacteria, antibacterial and antifungal activity, decontamination of mycotoxins	Vinderola and Ritieni (2015) and Luz et al. (2018)
	*L. buchneri*	Synthesis and/or metabolize 1,2-propanediol, improvement of aerobic stability, increasing the biogas yield	Zhang et al. (2009), Herrmann et al. (2015), Comino et al. (2014) and Zielińska et al. (2016)
	*L. diolivorans*	Metabolize of 1,2-propanediol to propionic acid, improving: aerobic stability of silage and biogas yield	Krooneman et al. (2002), Charley and Kung (2005) and Zielińska et al. (2017)
*Pediococcus*	*P. pentosaceus*	Probiotic bacteria, pediocin producers, increasing the preservation of dry matter and/or protein in silage, decontamination of mycotoxins	Dellaglio and Felis (2005) and Porto et al. (2017)
	*P. acidilactici*		
*Enterococcus*	*E. faecium*	Dynamic increase of bacterial population, decreasing pH in silage, probiotic activity	Dellaglio and Felis (2005) and Li and Nishino (2011)

Select LAB species most commonly occur in silage inoculants; the presented abilities of bacteria refers to specific strains not enumerated in the table

On the basis of the fermentative pathway, LAB are divided into three groups: homofermentative, relatively heterofermentative and heterofermentative. The group of homofermentative bacteria includes such species as: *P. damnosus* and *L. ruminis*. The group of relatively heterofermentative bacteria includes: *L. plantarum*,* L. pentosus*,* P. acidilactici*,* P. pentosaceus* and *E. faecium*. Heterofermentative bacteria include species of *Leuconostoc* spp., as well as some lactobacilli, such as *L. buchneri* (Dellaglio and Felis 2005).

Bacterial inoculants are produced by cultivation of bacterial strains according to optimal growth conditions, and the biomass is dried by lyophilization. The inoculants are commercially available in the form of lyophilizates with a content of about 95% of dry matter, which contains a mixture of live LAB cells designed to stimulate the ensiling of a given group of plants. In 1 g of the preparation, the number of units forming bacterial colonies is between 1 × 10^10^ and 5 × 10^11^. The preparation is given to plants after harvesting by spraying with an aqueous suspension of bacteria, used in a dose providing the number of bacterial cells intended for ensiling, which is a minimum 1 × 10^5^ CFU/g of plant material (Zielińska et al. 2015). The preservation of roughages by ensiling is most often carried out in tanks specially designed for this purpose: silos, ground heaps, bales sealed in polywrap or foil sleeves (Pahlow et al. 2003).

LAB inoculants are being readily used by farmers as part of sustainable agriculture and are applied in many modern day agriculture concepts, like organic farming, biofertilizers and biocontrol agents. The current market demand for inoculants in agriculture is reported to be $268.1 million and is slated to grow during the 2015–2020 forecast period due to high cost and demand for basic agrochemicals, like pesticide and fertilizer, as well as the popularity of ecological farming and the need for higher food production efficiency (Research and Markets Report 2016). European countries are leading the market with a share of 44% of the global silage additives market; rise in awareness for animal health is the major driving factor for the market. The Asia–Pacific silage additive market is expected to rise. According to Mordor Intelligence research and analysis, a majority of the global market share is concentrated between six major players: BASF, Lallemand, ForFarmers, Volac, CHR Hansen and Cargill (Mordor Intelligence Report 2017). In Europe, DeLaval, Pioneer, Blattin, Sano and Schaumann are companies that also possess a significant part of the marker. The most frequently mentioned species are *L. plantarum*,* L. buchneri*,* E. faecium* and *P.acidilactici*, and less often *L. rhamnosus* and *Lactococcus lactis*. The key companies in the market are expanding the business units in various geographical areas, focusing on agreements and partnerships with local players and distributors, as well as introducing new effective products through investments in R&D (Research and Markets Report 2016; Zielińska et al. 2015).

## Prevention of fungal growth and detoxification of mycotoxins by LAB inoculants

The modern approach of inhibiting the development of mould microflora in fodder involves the use of natural abilities of microorganisms for antagonistic interaction among themselves, and in particular, scientific attention is paid to the diverse species of LAB. LAB produce numerous compounds with antibacterial activity and inhibit the growth of fungi of the genera *Monilia*,* Aspergillus*,* Penicillium* and *Fusarium*. The production of fungistatic ingredients depends on the growth phase, which is the highest in the logarithmic growth phase, as well as on temperature, substrate, nutrients and pH (Dalie et al. 2010). According to Magnusson and Schnurer (2005), there are three mechanisms that explain the antimicrobial activity of LAB strains: production of organic acids, competition for nutrients and synthesis of antagonistic compounds. In addition, there are several scientific reports on peptides with fungistatic activity produced by LAB. Cyclic peptides composed mainly of proline and phenylalanine, glycine and l-leucine, as well as mevalonolactone, inhibit mould growth at concentrations of mg/cm^3^, but their antifungal properties are weaker than those of fatty acids (Ström et al. 2002; Magnusson and Schnurer 2005).

LAB strains isolated from plant environments are able to eliminate mycotoxins, such as OTA, patulin and AFs, and fusarium toxins like deoxynivalenol, zearalenone and fumonisins (Fuchs et al. 2008; Niderkorn et al. 2007). Strains that are characterized by the same sensitivity to mycotoxins may differ significantly in the degree of its degradation in the environment (Luz et al. 2018).

The effects of the synergistic action of the discussed group of microorganisms was confirmed by few model tests (Vinderola and Ritieni 2015). Several other studies have found that strains of the genus *Lactobacillus*, especially *L. plantarum* and *L. rhamnosus*, produce metabolites with a strong antifungal effect and demonstrate specific AFB_1_ removal, such as from fermented roughages. The degree of biodegradation seems to be dependent on the temperature and the initial inoculum (Ogunbanwo et al. 2005).

One of the global research trends is the attempt to limit the absorption of mycotoxins from the gastrointestinal tract of animals using preparations consisting of probiotic LAB strains. So far, these trials are in the research phase, although there are reports of reductions in AFB_1_ in the gastrointestinal tract of chickens (El-Nezami et al. 2000). The use of feed contaminated with fungi mixed with bacterial cultures isolated from the gastrointestinal tract of poultry reduced deoxynivalenol by 55%. Two other lactobacilli strains in the form of a probiotic preparation reduced the absorption of AFB_1_. The beneficial effect of bacteria is explained by increasing the amount of enzymes in food, which facilitates digestion and chelating of mycotoxins (El-Nezami et al. 2006).

The latest in vitro studies on the ability to bind AFB_1_ by lactobacilli probiotic strains (including commercially available *L. rhamnosus* GG and *L. casei* Shirota, *P. freudenreichii* ssp., *shermanii* JS and *E. coli*) showed that mycotoxin binding was fast (removal from the supernatant was already observed by time zero of contact) and stable (almost no differences in removal were observed after 72 h of contact of the strains with AFB_1_) (Vinderola and Ritieni 2015). Recently, Luz et al. (2018) studied the influence of some *Bifidobacterium* spp. and *Lactobacillus* spp. on OTA reduction in medium by adsorption to the cell wall, as well as hydrolytic degradation of mycotoxin by bacterial proteases. The strains that evidenced the highest OTA biodegradation were claimed to be used in the silage industry as inoculants or as feed additives to reduce the problem of the intake of OTA in animal production, considering these animals are the most susceptible livestock to the toxic effects of OTA.

Unfortunately, there are only a few reports attempting to evaluate LAB capability of mycotoxin detoxification under production conditions. One of the latest examples is a preparation based on a strain of *L. plantarum* S KKP 2021 p with the ability to eliminate OTA, which was used for ensiling of roughages contaminated with the mycotoxin. After the fermentation of meadow sward and maize plants, with the participation of the bacterial inoculant of the new strain, the content of OTA was reduced by more than 80%, relative to its content in the starting material (Zielińska et al. 2014).

## Other trends in process of plant fermentation involving LAB

The new generation of bacterial inoculants should combine the synergistic action of LAB strains that ensure microbiological safety of silages made from meadow sward, alfalfa, legumes, maize plants and grains, as well as cereals and waste products of the food industry. At the same time roughages should be characterized by high oxygen stability lasting for more than 1 year of storage (Zhang et al. 2009). The ability to inhibit the growth of pathogenic bacteria is represented by certain strains of the species: *L. lactis*,* Streptococcus lactis*,* L. acidophilus*,* L plantarum*, *L. brevis* or *L. buchneri* and may be a result of the synergistic action of the produced metabolites: lactic acid, acetic acid, hydrogen peroxide, lactate peroxidase, lysozyme, bacteriocins and propylene glycol. However, the synthesis of bacteriocins and/or bacteriocin-like substances has also been reported to account for the antagonistic activity exerted by lactobacilli (Magnusson and Schnurer 2005).

The influence of the starter culture containing LAB strains of *L. plantarum* and *L. buchneri* have been demonstrated in the example of alfalfa silage prepared with or without the addition of selected bacterial strains, with a proven ability to control undesirable bacteria that are potentially pathogenic to animals. The application of bacterial inoculants in alfalfa ensiling resulted in a significant reduction of the total number of undesirable microorganisms, including *Listeria* spp. and *Cl. perfringens*. Additionally, silage treatment with the LAB preparations resulted in the complete elimination of pathogenic bacteria of the genus *Salmonella* and *E. coli* bacteria (Zielińska et al. 2015). Silages that are characterized by low clostridial contamination and by low amount of yeast are aerobically stable for a longer period of time (Muck et al. 2018).

When targeting silage for biogas production, in addition to the microbiological quality and stability of functional characteristics, the efficiency of biogas from dry matter of organic silage is an important quality factor. Under the influence of inoculants containing selected bacterial strains from *L. buchneri* and *L. diolivorans* species, biochemical changes may be aimed at limiting the synthesis of lactic acid, while increasing the synthesis of acetic and propionic acids, and thus, ensuring overall stability, aerobic stability and increased biogas yield from these silages (Herrmann et al. 2015; Nussbaum 2012).

In studies investigating the impact of LAB inoculants on the improvement of microbiological quality and inhibition of the growth of pathogenic microorganisms in silages, new genetic techniques have been used, allowing the differentiation of strains. PCR and its modification, polymerase chain reaction-denaturing gradient gel electrophoresis (PCR-DGGE), enables obtaining information on the genotypes of microorganisms based on the analysis of differences in selected parts of the genome. In this way, changes in the species of microorganisms can be studied during ensiling. Moreover, length heterogeneity PCR (LH-PCR) uses variation in the length of a particular gene between different microbial species to determine how many species may be active in the environment (Muck 2013; Garofalo et al. 2017).

Zhou et al. (2016) considered fingerprint techniques such as DGGE as interesting approaches to investigate the microbial population dynamics during the ensiling process, e.g., the influence of low temperatures that contribute to creating the various evolution of the LAB population responsible for whole plant corn silage fermentation. Zielińska et al. (2015) acknowledged the usefulness of RAPD-PCR technique (random amplification of polymorphic DNA) to evaluate the genetic diversity of LAB strains used in silage inoculants.

High-throughput sequencing technology revealed the diversity of the bacterial community in maize and alfalfa silages. The bacterial community profiles varied during ensiling and were influenced by aerobic exposure; nevertheless, *Lactobacillus* spp. dominated during the ensiling process. The results of those papers provided valuable information for the further development and application of bacteria in silage, although the underlying relationship between dynamic patterns of silage microbiota and the outcome of fermentation needs further extensive studies (Zhou et al. 2016; Hu et al. 2018; Ogunade et al. 2018).

Guo et al. (2018) studied the effect of the inoculants *L. plantarum* and *L. buchneri* on the microbiological quality in ensiled *Medicago sativa* and profiled metabolome and bacterial community dynamics using molecular biology methods based on small molecule real-time sequencing technology (SMRT). Metabolomic profiling analysis provided a deep insight into metabolites in silages. Moreover, the SMRT method revealed the microbial composition and its succession during the ensiling process and species level.

## Summary

LAB have been used for centuries in the production of fermented food and feed due to its ability to use sugars for the synthesis of lactic acid, thereby reducing the pH and protecting against the development of undesirable microflora. Moreover, these microorganisms have the GRAS (generally recognized as safe) status. The tradition of using LAB and the current knowledge about the positive effects of those species on human and animal health as potential probiotic organisms, give hope for their use as alternative methods of preservation of food and feed products that meet the requirements of today’s consumers, farmers and food and feed companies.

While the inhibition of pathogenic microflora is a trend in the use of LAB inoculants for silages, for years it has still been explored by scientists and feed companies. It is worth noting that their use against the presence of toxic secondary metabolites of filamentous fungi is still not used much. Inhibition of mould growth in many model studies points to the complex interaction between many metabolites of LAB, including those still unknown, which still poses new challenges for researchers on this subject and still raises new possibilities for the application of this group of microorganisms. Previous studies on the possibility of reducing toxicity, e.g., AF adsorption in the gastrointestinal tract, are ambiguous, and it is difficult to assess the future usefulness of the discussed group of microorganisms in this process. The available data, however, allows the positive assessment of LABs with the ability to decontaminate mycotoxins as starter cultures in the process of ensiling feed that increase the quality of roughages.

The use of starter cultures of LAB strains characterized by the ability to lower the level of pathogenic microorganism may be an optimal method of forage preservation with reference to the most hazardous species, like *E. coli*,* Salmonella* spp. or *L. monocytogenes*. Still, some limitations refers to the not fully revealed mechanisms of interactions between LAB cells and pathogens.

In the past, silage studies had been intensively focusing on chemical analyses and aerobic stability research, while microbial population and their dynamic changes have only been analysed to a minor extent. Today, we have an insight into the main fermentation process, thanks to molecular biology techniques; however, to fully understand the process, we need to obtain a more complete picture of the microbiological flora during the ensiling process. New, powerful methods within the field of DNA that were discussed herein have evolved over the past two decades and might bring some new facts, allowing more targeted usage of bacterial inoculants intended for ensiling. The new decade of silage studies should be occupied by research on metagenomic analyses and metabolomic studies.

## References

[CR1] Ben-Dov E, Shapiro OH, Siboni N, Kushmaro A (2006). Advantage of using inosine at the 3′termini of 16S rRNA gene universal primers for the study of microbial diversity. Appl Environ Microbiol.

[CR2] Broberg A, Jacobsson K, Ström K, Schnürer J (2007). Metabolite profiles of lactic acid bacteria in grass silage. Appl Environ Microbiol.

[CR3] Brusetti L, Borin S, Mora D, Rizzi A, Raddadi N, Sorlini C, Daffonchio D (2006). Usefulness of length heterogeneity-PCR for monitoring lactic acid bacteria succession during maize ensiling. FEMS Microbiol Ecol.

[CR4] Charley R, Kung L (2005) Treatment of silage with *Lactobacillus diolivorans*. US Patent US20050281917 A1

[CR5] Coffey R, Cummins E, Ward S (2009). Exposure assessment of mycotoxins in dairy milk. Food Control.

[CR6] Comino L, Tabacco E, Righi F, Revello-Chion A, Quarantelli A, Borreani G (2014). Effects of an inoculant containing a *Lactobacillus buchneri* that produces ferulate-esterase on fermentation products, aerobic stability, and fibre digestibility of maize silage harvested at different stages of maturity. Anim Feed Sci Technol.

[CR7] Dalie DKD, Deschamps AM, Richard-Forget F (2010). Lactic acid bacteria—potential for control of mould growth and mycotoxin. A review. Food Control.

[CR8] Dellaglio F, Felis GE (2005). Probiotics scientific aspects.

[CR9] Doi K, Nishizaki Y, Fujino Y, Ohshima T, Ohmomo S, Ogata S (2009). *Pediococcus lolii* sp. nov., isolated from ryegrass silage. Int J Syst Evol Microbiol.

[CR10] Edrington TS, Fox WE, Callaway TR, Anderson RC, Hoffman DW, Nisbet DJ (2009). Pathogen prevalence and influence of composted dairy manure application on antimicrobial resistance profiles of commensal soil bacteria. Foodborne Pathog Dis.

[CR11] El-Nezami HS, Mykkanen H, Kankaanpää P, Salminen S, Ahokas J (2000). Ability of *Lactobacillus* and *Propionibacterium* strains to remove aflatoxin B_1_ from the chicken duodenum. J Food Prot.

[CR12] El-Nezami HS, Polychronaki NN, Ma J, Zhu H, Ling W, Salminen EK, Juvonen RO, Salminen SJ (2006). Probiotic supplementation reduces a biomarker for increased risk of liver cancer in young men from southern China. Am J Clin Nutr.

[CR13] Fuchs S, Sontag G, Stidl R, Ehrlich V, Kundi M, Knasmüller S (2008). Detoxification of patulin and ochratoxin A, two abundant mycotoxins, by lactic acid bacteria. Food Chem Toxicol.

[CR14] Garofalo C, Bancalari E, Milanović V, Cardinali F, Osimani A, Sardaro MLS, Bottari B, Bernini V, Aquilanti L, Clementini F, Neviani E, Gatti M (2017). Study of the bacterial diversity of foods: PCR-DGGE versus LH-PCR. Int. J Food Microbiol.

[CR15] Guo XS, Ke WC, Ding WR, Ding LM, Xu DM, Wang WW, Zhang P, Yang FY (2018). Profiling of metabolome and bacterial community dynamics in ensiled *Medicago sativa* inoculated without or with *Lactobacillus plantarum* or *Lactobacillus buchneri*. Sci Rep.

[CR16] Herrmann C, Idler C, Heirmann M (2015). Improving aerobic stability and biogas production of maize silage using silage additives. Bioresour Technol.

[CR17] Holley RA, Arrus KM, Omiński KH, Tenuta M, Blank G (2006). *Salmonella* survival in manure-treated soils during simulated seasonal temperature exposure. J Environ Qual.

[CR18] Hu Z, Chang J, Yu J, Li S, Niu H (2018). Diversity of bacterial community during ensiling and subsequent exposure to air in whole-plant maize silage. Asian–Australas J Anim Sci.

[CR19] Krooneman J, Faber F, Alderkamp AC, Qude Elferink SJKW, Driehuis F, Cleenwerck I, Swings J, Gottschal JC (2002). *Lactobacillus diolivorans* sp. nov., a 1,2-propanediol-degrading bacterium isolated from aerobically stable maize silage. Int J Syst Evol Microbiol.

[CR20] Li Y, Nishino N (2011). Monitoring the bacterial community of maize silage stored in bunker silo inoculated with *Enterococcus**faecium*,* Lactobacillus plantarum* and *Lactobacillus buchneri*. J App Microbiol.

[CR21] Luz C, Ferrer J, Manes J, Meca G (2018). Toxicity reduction of ochratoxin A by lactic acid bacteria. Food Chem Toxicol.

[CR22] Magnusson J, Schnurer J (2005). Antifungal lactic acid bacteria as biopreservatives. Trends Food Sci Technol.

[CR23] Muck RE (2013). Recent advances in silage microbiology. Agric Food Sci.

[CR24] Muck RE, Nadeau EMG, McAllister TA, Contreras-Govea MC, Santos MC, Kung L (2018). Silage review: recent advances and future uses of silage additives. J Dairy Sci.

[CR25] Müller T, Ulrich A, Ott EM, Müller M (2001). Identification of plant-associated enterococci. J Appl Microbiol.

[CR26] Niderkorn V, Morgavi DP, Pujos E, Tissandier A, Boudra H (2007). Screening of fermentative bacteria for their ability to bind and biotransform deoxynivalenol, zearalenone and fumonisins in an in vitro simulated corn silage model. Food Addit Contam.

[CR27] Nussbaum H (2012) Effects of silage additives based of homo- or heterofermentative lactic acid bacteria on methane yields in the biogas processing In: Proceedings of the XVI international silage conference, 2–4 July 2012, Hameenlinna, Finland. pp 452–453

[CR28] O’Brien M, O’Kiely P, Forristal P, Fuller H (2005). Fungi isolated from contaminated baled grass silage on farms Irish Midlands. FEMS Microbiol Lett.

[CR29] O’Brien M, Nielsen KF, O’Kiely P, Forristal PD, Fuller HT, Frisvad JC (2006). Mycotoxins and other secondary metabolites produced in vitro by *Penicillium paneum* Frisvad and *Penicillium roqueforti* Thom isolated from baled grass silage in Ireland. J Agric Food Chem.

[CR30] O’Brien M, O’Kiely P, Forristal PD, Fuller HT (2007). Quantification and identification of fungal propagules in well-managed baled grass silage and in normal on-farm produced bales. Anim Feed Sci Technol.

[CR31] Ogunade IM, Jiang Y, Pech Cervantes AA, Kim DH, Oliveira AS, Vyas D, Weinberg ZG, Jeong KC, Adesogan AT (2018). Bacterial diversity and composition of alfalfa silage as analyzed by IIIumina MiSeq sequencing: effects of *Escherichia coli* 0157:H7 and silage additives. J Dairy Sci.

[CR32] Ogunbanwo ST, Enitan AM, Emeya P, Okanlawon BM (2005). Influence of lactic acid bacteria on fungal growth and aflatoxin production in ogi, an indigenous fermented food. Adv Food Sci.

[CR33] Fernandes Oliveira Carlos Augusto, Bovo Fernanda, Humberto Carlos, Vincenzi Alessandra, Ravindranadha Kasa (2013). Recent Trends in Microbiological Decontamination of Aflatoxins in Foodstuffs. Aflatoxins - Recent Advances and Future Prospects.

[CR34] Ongeng D, Geeraerd AH, Springael D, Ryckeboer J, Muyanja C, Mauriello G (2015). Fate of *Escherichia coli* O157:H7 and *Salmonella enterica* in the manure-amended soil–plant ecosystem of fresh vegetable crops: a review. Crit Rev Microbiol.

[CR35] Pahlow G, Muck RE, Driehuis F, Elferink SJWHO, Spoelstra SF, Buxton DR, Muck RE, Harrison JH (2003). Microbiology of ensiling. Silage science and technology.

[CR36] Pang H, Zhang M, Qin G, Tan Z, Li Z, Cai Y (2011). Identification of lactic acid bacteria isolated from corn stovers. Anim Sci J.

[CR37] Pang H, Qin G, Tan Z, Li Z, Wang Y, Cai Y (2011). Natural populations of lactic acid bacteria associated with silage fermentation as determined by phenotype, 16S ribosomal RNA and recA gene analysis. Syst Appl Microbiol.

[CR38] Pauly TM, Rodhe I (2002) Slurry application on ley-effect of method on the hygienic quality of grass silage In: Proceedings of the XIII International Silage Conference, Auchincruive, Scotland, pp. 410–411.

[CR39] Porto MC, Kuniyoshi TM, Azevedo PO, Vitolo M, Oliveira RP (2017). *Pediococcus* spp: an important genus of lactic acid bacteria and pediocin producers. Biotechnol Adv.

[CR40] Mordor Intelligence Report (2017) Silage additive market—growth, industry analysis and forecasts (2017–2020). https://www.mordorintelligence.com/industry-reports/global-silage-additives-market-industry. Accessed 22 Aug 2018

[CR41] Research and Markets Report (2016) Global agricultural inoculants market-by type, microbes, mode of application, application and geography market shares, forecasts and trends (2015–2020). https://www.researchandmarkets.com/reports/3786558/global-agricultural-inoculants-market-research. Accessed 21 Aug 2018

[CR42] Richard E, Heutte N, Sage L, Pottier D, Bouchart V, Lebailly P, Garon D (2007). Toxigenic fungi and mycotoxins in mature corn silage. Food Chem Toxicol.

[CR43] Rubert J, León N, Sáez C, Martins CPB, Godula M, Yusà V, Mañes J, Soriano MJ, Soler C (2014). Evaluation of mycotoxins and their metabolites in human breast milk using liquid chromatography coupled to high resolution mass spectrometry. Anal Chim Acta.

[CR44] Ström K, Sjögren J, Broberg A, Schnürer J (2002). *Lactobacillus plantarum* MiLAB 393 produces the antifungal cyclic dipeptides cyclo(l-Phe-l-Pro) and cyclo(l-Phe-*trans*-4-OH-l-Pro) and phenyl lactic acid. Appl Environ Microbiol.

[CR45] Van Egmond HP (2004). Natural toxins: risks, regulations and the analytical situation in Europe. Anal Bioanal Chem.

[CR46] Vinderola G, Ritieni A (2015). Role of probiotics against mycotoxins and their deleterious effects. J Food Res.

[CR47] Wang L-T, Kuo H-P, Wu Y-C, Tai CJ, Lee FL (2009). *Lactobacillus taiwanensis* sp. nov., isolated from silage. Int J Syst Evol Microbiol.

[CR48] Weinberg ZG, Muck RE, Weimer PJ (2003). The survival of silage inoculant lactic acid bacteria in rumen fluid. J Appl Microbiol.

[CR49] You Y, Rankin SC, Aceto HW, Benson CE, Toth JD, Dou Z (2006). Survival of *Salmonella enterica* serovar Newport in manure and manure-amended soils. Appl Environ Microbiol.

[CR50] Zhang T, Li L, Wang XF, Zeng ZH, Hu YG, Cui ZJ (2009). Effects of *Lactobacillus buchneri* and *Lactobacillus plantarum* on fermentation, aerobic stability, bacteria diversity and ruminal degradability of alfalfa silage. World J Microbiol Biotechnol.

[CR51] Zhou Y, Drouin P, Lafreniere C (2016). Effect of temperature (5–25 °C) on epiphytic lactic acid bacteria populations and fermentation of whole-plant corn silage. J Appl Microbiol.

[CR52] Zielińska KJ, Stecka KM, Kapturowska AU, Kupryś MP, Miecznikowski AH (2014) Strain of *Lactobacillus plantarum* S, the use of the strain of *Lactobacillus plantarum* and the preparation for roughages ensiling. US Patent 8.697.423

[CR53] Zielińska K, Fabiszewska A, Stefańska I (2015). Different aspects of *Lactobacillus* inoculants on the improvement of quality and safety of alfalfa silage. Chil J Agric Res.

[CR54] Zielińska K, Fabiszewska A, Stecka K, Świątek M (2016) Strain *Lactobacillus buchneri* A, composition, a multi component preparation for starch rich plant preservation, their use and a method for plant preservation. US Patent 9.370.199

[CR55] Zielińska K, Fabiszewska A, Świątek M, Szymanowska-Powałowska D (2017). Evaluation of the ability to metabolize 1,2-propanediol by heterofermentative bacteria of the genus *Lactobacillus*. Electron J Biotechnol.

